# Replantation of Immature Avulsed Teeth with Prolonged Extraoral Dry Storage: A Case Report

**DOI:** 10.5005/jp-journals-10005-1137

**Published:** 2012-02-24

**Authors:** Shweta Jain, Vijay Agarwal, Arun Kumar Gupta, Pramod Prabhakar

**Affiliations:** Senior Lecturer, Department of Conservative Dentistry and Endodontics NIMS Dental College, NIMS University, Jaipur, Rajasthan, India; Senior Lecturer, Department of Orthodontics, NIMS Dental College NIMS University, Jaipur, Rajasthan, India; Professor and Head, Department of Prosthodontics, NIMS Dental College, NIMS University, Jaipur, Rajasthan, India; Associate Professor, Department of Prosthodontics, NIMS Dental College, NIMS University, Jaipur, Rajasthan, India

**Keywords:** Replantation, Avulsion, Replacement resorption

## Abstract

This case report presents delayed replantation of avulsed teeth after extended extraoral period and nonphysiological storage. Yet, long-term prognosis is not good, it presents alternate treatment modality to immediately restore esthetic and function as well as to promote the growth of alveolar crest for proper eruption of adjacent unaffected teeth until a definite prosthetic treatment seems appropriate.

**How to cite this article:** Jain S, Agarwal V, Gupta AK, Prabhakar P. Replantation of Immature Avulsed Teeth with Prolonged Extraoral Dry Storage: A Case Report. Int J Clin Pediatr Dent 2012;5(1):68-71.

## INTRODUCTION

Avulsion is defined as total displacement of the tooth out of its alveolar socket or exarticulation. It occurs most often in children 7 to 9 years old, an age when the relatively resilient alveolar bone provides only minimal resistance to extrusive forces and the maxillary central incisors are the teeth most commonly affected. Avulsion accounts for 0.5 to 16% of traumatic injuries in the permanent dentition.^1^

Management of avulsion in mixed dentition period often presents a challenge due to continued alveolar growth of maxilla and mandible, incomplete root development, difficult child behavior management, delayed reporting to the dental offices and nonphysiological storage of avulsed tooth. Replantation is the recommended procedure and refers to the insertion and temporary fixation of completely or partially avulsed teeth that have resulted from traumatic injury.

Replantation of an avulsed tooth depends on physiological status of periodontal ligament (PDL), the stage of root development and the length of extraoral time. The prognosis is best for teeth replanted within 5 minutes after avulsion.^2^ Healing with a normal periodontal ligament (i.e. regeneration) after replantation will occur only if the innermost cell layers along the root surface are vital.^1^ On an average these teeth are functional for 5 years ^3^ and most are ultimately lost because of progressive replacement root resorption or external inflammatory root resorption. Andreason has reported that if the tooth has been out of the mouth for more than 2 hours, there is a 95% chance of external resorption.^4^ However, if managed properly, avulsed teeth with a vital periodontal ligament can be replanted and will remain functional for some years.^5^This case report describes an emergency dental approach after a traumatic dentoalveolar injury to the mixed dentition. Avulsed maxillary incisors were promptly managed in emergency visit in order to maintain esthetic and function on immediate basis. Further intervention will be followed by the outcome of the treatment and evaluation of prognosis on periodic recall basis.

## CASE REPORT

A 9-year-old boy came to dental office in morning session, had slipped and fallen at a playground previous evening. His two front teeth were avulsed and stored in a polyethylene bag. The patient’s medical history was noncontributory. On examination, the patient did not show any signs or symptoms of neurological or extraoral injury. Intraoral examination revealed mixed dentition with missing permanent maxillary right central and lateral incisors (Fig. 1A). He showed incisal overjet of 3.5 mm and end-to-end molar relationships. Oral hygiene was fair. No other oral injury was detected clinically. Adjacent teeth elicited positive response to vitality test. Examination of the avulsed tooth revealed that the crowns were intact and that the roots of incisors had wide apical foramina (Fig. 1B). Preoperative radiograph revealed no other hard-tissue injury (Fig. 1C). On examination of avulsed teeth necrotic dried remnants of periodontal tissue were present over the roots. The avulsed teeth had been kept dry for about 16 hours.

**Fig. 1A:**
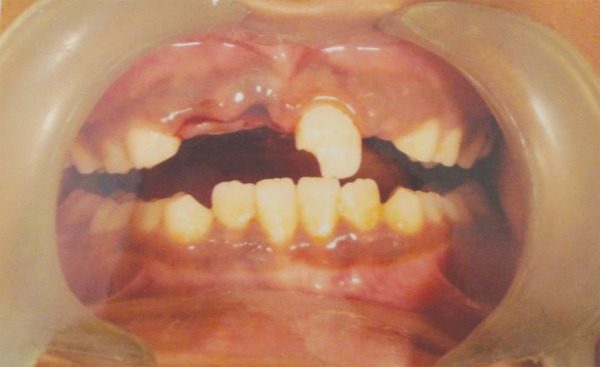
Preoperative intraoral photograph with missing teeth

**Fig. 1B:**
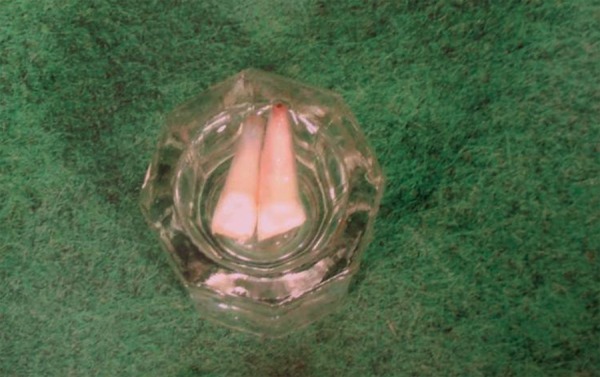
Avulsed teeth

**Fig. 1C:**
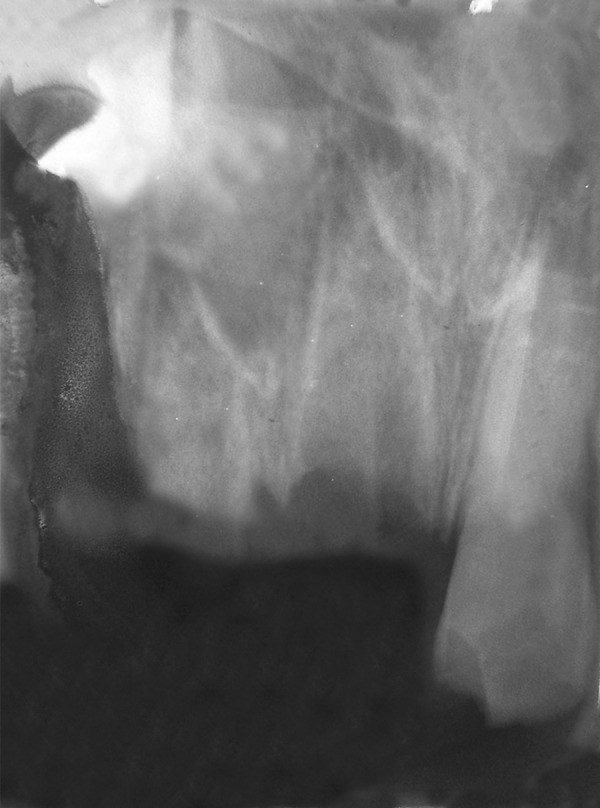
Preoperative intraoral radiograph showing empty alveolar sockets

The available treatment options were explained to the parents and it was decided to replant the avulsed incisors. The roots of the avulsed teeth were scraped gently to remove the necrotic periodontal tissue and access cavities prepared through crowns of avulsed teeth. Thorough biomechanical preparation was done with hand K-files (Dentsply, Maillefer, Ballaigues, Switzerland) and 2.5% sodium hypochlorite solution and normal saline solution were used as root canal irrigants. Roots were dried with absorbent points and obturated with gutta-percha points (Dentsply, Maillefer, Ballaigues, Switzerland) and zinc oxide eugenol-based sealer. Root end cavities of around 3 mm were prepared and restored with intermediate restorative material (IRM, Dentsply). After initial setting of IRM, teeth were placed in tetracycline solution for around 20 minutes.

Following local anesthesia with 2% lidocaine containing 1:20,000 epinephrine (Xylocaine; AstraZeneca Pharma Ind Ltd, Bangaluru, India) sockets were explored gently for the presence of tooth or alveolar bone fragments. Fresh bleeding was established in the alveolar sockets, teeth were replanted and splinted to the adjacent teeth with acid etch resin and wire splint. A 7-day course of systemic penicillin and analgesic were prescribed; antitetanus booster was given to prevent any systemic complication. Another radiograph was obtained to confirm proper positioning of the replanted incisors (Fig. 1D). Splint was removed after 14 days. The patient was seen again at 2 weeks, 6 weeks (Fig. 1E) and 12 weeks after replantation and then followed half-yearly. At the time of writing, the patient had been followed for 2 years (Figs 1F and G). Esthetic correction of teeth was done with direct composite resin. Full coverage crowns with affected incisors and periodic pulp vitality testing with adjacent unaffected teeth was advised. The replanted incisors developed mild replacement root resorption. Nevertheless, they remained functional and were esthetically acceptable. The patient and his parent were informed about ankylosis or replacement resorption as the patient grew. Long-term treatment might need sacrifice of teeth and prosthodontic replacement.

**Fig. 1D:**
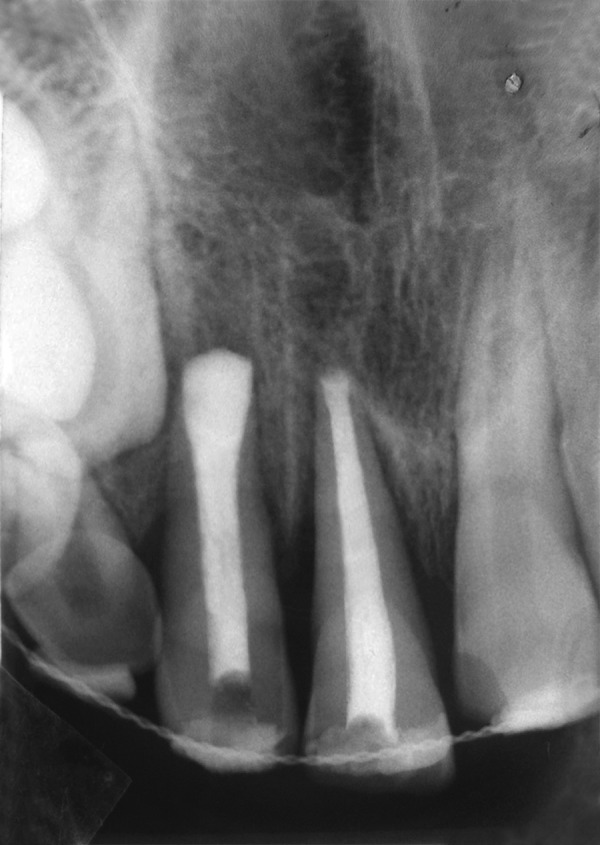
Immediate postoperative radiograph with splint in place

**Fig. 1E:**
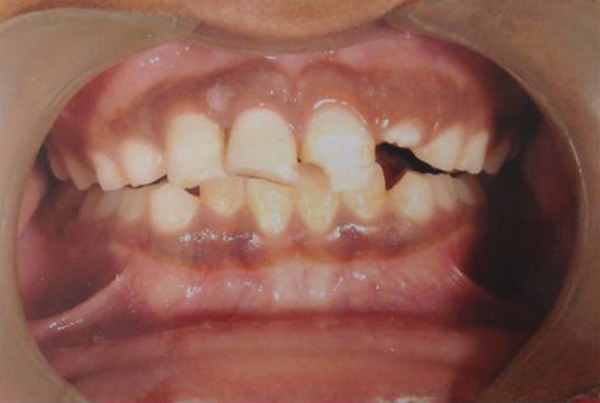
Six weeks follow-up intraoral photograph of stabilized teeth

**Fig. 1F:**
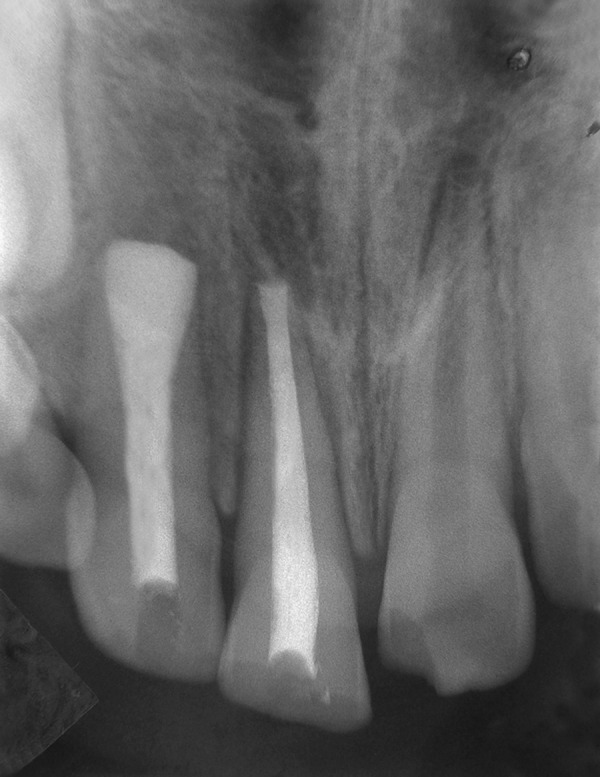
Two years follow-up intraoral radiograph

**Fig. 1G:**
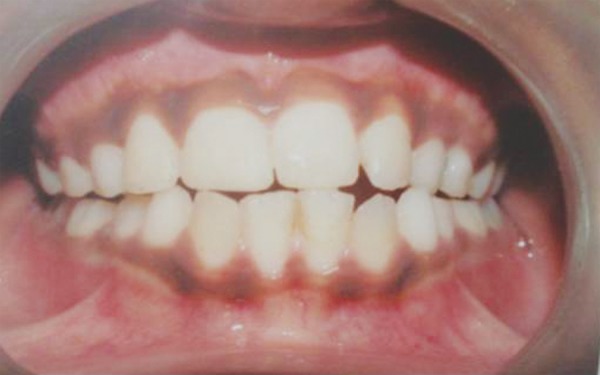
Two years follow-up intraoral photograph

## DISCUSSION

According to the International Association of Dental Traumatology (IADT) 2007 replantation of avulsed permanent tooth with open apex with extraoral time more than 60 minutes is not indicated.^6^ As the patient was very young and missing anterior teeth have a negative effect on physical attractiveness of the person; other prosthetic solutions were not found to be suitable, so decision of replantation of avulsed teeth was made with the consent of parents. If the tooth remained dry for more than 60 minutes no consideration should be given to preserve the periodontal ligament and endodontic therapy could be performed extraorally.^7^ Given that replacement root resorption was inevitable after the prolonged period of dry storage, it was thought that further drying and handling of the root surface was unlikely to worsen the prognosis, so the avulsed incisors were obturated extraorally. Root ends were filled with IRM to achieve better apical seal.

Chemical treatment of root surface with various agents, such as tetracycline before replantation has been suggested in the hope of slowing down the resorption process. After removal of necrotic periodontal ligaments, such roots should be chemically treated with 2.4% acidulated sodium fluoride solution (pH 5.5) for 20 minutes or in tetracycline before replantation.^8^ In present case avulsed incisors were chemically treated with tetracycline. So, in this case the appropriate treatment was performed. The replanted tooth should be splinted flexibly to the adjacent teeth for 7 to 10 days to enhance periodontal healing. Systemic antibiotics are often recommended after replantation, but their effectiveness in preventing root resorption is questionable.^9^

Systemic and/or topical application of different medicaments is generally used as antiresorption regeneration therapy (ART)^10^ to depress resorption activity and support regeneration in the PDL. An enamel matrix derivative gel (Emdogain; Biora AB, Malmo, Sweden) has shown to be useful in enhancing periodontal healing in teeth for which extra-alveolar storage has been extended, as it has the potential to promote regeneration of periodontal ligament from the socket-side periodontal cell populations.^11^ Use of drugs like doxycycline (topical and systemic),^10^minocycline,^12^ bisphosphonates (e.g. zoledronate, etidronate, alendronate),^13^ dexamethasone,^14^ topical application of propolis^15^ may decrease or prevent inflammatory root resorption and replacement root resorption and facilitates the regeneration of periodontal tissues after replantation. Recently a low-level laser therapy^16^ is also advocated to promote favorable healing in cases of delayed replantation. However, the use of these agents in replantation is still experimental and more data to support clinical effectiveness are required.

As the evidence supports the importance of immediate replantation, parents, sport coaches and first aid caregivers should be trained to replant teeth immediately at the scene of an accident through direct education or technique demonstration posters. Properly fitted mouthguards can reduce the severity of dental injury, so dentists can educate all members of sport team to provide first aid care for avulsed tooth and to encourage the use of custom mouthguards during sports.^17^

Teeth replanted after 60 minutes of dry storage become ankylosed and are resorbed within 7 years in young patients, whereas teeth replanted under similar conditions in patients older than 16 years may remain functional for considerably longer periods. More rapid resorption of teeth in children is related to the higher rate of bone remodeling in children than in adults.^18^ Teeth replanted from 6 to 48 hours after avulsion and treated endodontically are shown to be clinically functional for a number of years with the estimated mean survival time of 57.3 months^19^ to 5 years.^3^

Avulsion of the incisors in young patients may results in infraocclusion as the patients grow for which surgical technique of decoronation has recently suggested and recommended to preserve the contour of the alveolar ridge, and when the infraocclusion of the tooth crown is more than 1 mm.^20^ Many different alternative treatments are suggested in the literature, such as extraction and replacement by another tooth orthodontically, autotransplantation, implants or other prosthetic therapy^21^ but loss or extraction of teeth in a growing alveolar process will result in resorption of the crest, loss of development in that region and unilateral space closure. For this reason, extraction followed by fixed prostheses and implants should be avoided in early ages. Replantation usually not recommended, if the patient’s medical condition contraindicates replantation.

## CONCLUSION

Although the risk of progressive resorption and subsequent tooth loss is high, delayed replantation can restore the patient’s esthetic appearance and occlusal function shortly after the injury and the replanted incisors can remain functional for some years.
